# Obtaining a series of native gradient promoter-5′-UTR sequences in *Corynebacterium glutamicum* ATCC 13032

**DOI:** 10.1186/s12934-020-01376-3

**Published:** 2020-06-03

**Authors:** Ning Li, Weizhu Zeng, Sha Xu, Jingwen Zhou

**Affiliations:** 1 Key Laboratory of Industrial Biotechnology, Ministry of Education and School of Biotechnology, Jiangnan University, 1800 Lihu Road, Wuxi, 214122 Jiangsu China; 2 National Engineering Laboratory for Cereal Fermentation Technology, Jiangnan University, 1800 Lihu Road, Wuxi, 214122 Jiangsu China; 3 Jiangsu Provisional Research Center for Bioactive Product Processing Technology, Jiangnan University, 1800 Lihu Road, Wuxi, 214122 Jiangsu China

**Keywords:** *Corynebacterium glutamicum*, Amino acids, RNA-Seq, Promoter engineering, Fine-tuning, *mCherry*

## Abstract

**Background:**

*Corynebacterium glutamicum* is an important industrial microorganism used for the production of many valuable compounds, especially amino acids and their derivatives. For fine-tuning of metabolic pathways, synthetic biological tools are largely based on the rational application of promoters. However, the limited number of promoters make it difficult.

**Results:**

In this study, according to the analysis of RNA-Seq data, 90 DNA fragments with lengths of 200-500 bp that may contain promoter-5′-UTR (PUTR) sequences were amplified and linked to a fluorescent protein gene. When compared with the common strong PUTR P_sod_UTR, 17 strong PUTRs were obtained, which maintained stable expression strengths from the early to post stationary phase. Among them, P_NCgl1676_UTR was the strongest and its fluorescent protein expression level was more than five times higher than that of P_sod_UTR. Furthermore, nine typical chemicals related to the biosynthesis of sulfur-containing amino acids (such as l-methionine, l-cysteine) were selected as stress substances to preliminarily explore the stress on these PUTRs. The results showed that the expression of P_brnF_UTR was activated by l-methionine, while that of P_NCgl1202_UTR was severely inhibited by l-lysine.

**Conclusions:**

These findings demonstrated that the selected PUTRs can stably express different genes, such as the red fluorescence protein gene, and can be useful for fine-tuning regulation of metabolic networks in *C. glutamicum* or for establishing high-throughput screening strategies through biosensor for the production of useful compounds.

## Background

*Corynebacterium glutamicum* can biosynthesize many useful compounds from coarse feedstocks. However, it is impractical to directly use wild type strains for efficient biosynthesis of these compounds. Random mutagenesis or directional metabolic engineering of wild microorganisms are necessary to obtain high titer strains. Mutagenesis and screening of strains have resulted in efficient microbial production of many useful compounds, especially different amino acids [1–3]. As random mutagenesis may not always be successful in producing some new useful compounds [4], researchers prefer metabolic engineering and synthetic biology methods to engineer strains for the production of target products [5]. Remodeling the metabolic flux of the target strain can achieve efficient biosynthesis of many compounds. However, the imbalance between cell growth and target products biosynthesis becomes a bottleneck for further accumulation of target products [6, 7]. To solve this problem, the expression level of some genes needs to be regulated properly.

Since upregulation of gene expression at genome level by increasing the number of gene copies is inefficient, expression of genes on plasmids with high copy number is the most used strategy. However, this could increase the metabolic burden of bacteria [8, 9]. Besides, plasmid instability and antibiotics addition are also unfavorable for industrial applications [10]. Although gene expression can be regulated at the post-transcriptional level, it is still inadequate for practical applications owing to the lack of available methods. The most direct and convenient choice to control gene expression is at the transcription and translation level, which can be controlled by using promoters or PUTRs (promoter-5′-untranslated region) [11, 12]. By using PUTRs with different strengths, the fine-tuning of gene expression level can be achieved by replacing PUTRs of appropriate strength, ultimately alleviating the imbalance between cell growth and target product synthesis [13]. Therefore, more native PUTRs should be investigated to design complex genomic metabolic engineering strategies. Recently, many researchers have made significant efforts to obtain and engineer PUTRs. In addition to simply upregulating or downregulating gene expression by replacing PUTRs [14], some PUTRs can be further designed as biosensors to achieve more complicated goals [15–17]. These constructed PUTRs significantly enrich the toolbox for metabolic engineering of microorganisms for the biosynthesis of various useful compounds.

Many amino acids including l-cysteine can be produced by other microbes like *E. coli* according to the current available reports. However, *C. glutamicum* is still the mostly used microorganism to produce amino acids on industrial scale, mostly for three advantages compare to *E. coli*: (1) *C. glutamicum* has no endotoxins; (2) *C. glutamicum* could grow well on cheaper culture conditions; (3) Very rare reports about bacteriophage contamination about *C. glutamicum*. Compared with the various metabolic engineering toolboxes available for *E. coli*, *C. glutamicum* has very few toolboxes, which significantly hinders its use as a popular chassis microorganism. In recent times, increasing metabolic engineering toolboxes have been developed for *C. glutamicum*, such as *sacB* based counter selection [18], *upp* based counter-selection [19], Cre-loxP-mediated genome editing [20] and CRISPR-related editing system (CRISPRi [21], CRISPR-Cas9 [22–24], and CRISPR-Cpf1 [25, 26]). However, these knockout and knock-in tools cannot meet the requirements for fine-tuning regulation of metabolic pathway or network. Therefore, it is necessary to obtain more promoters or PUTRs for *C. glutamicum*. In addition to the most common inducible promoters (P_trc_-M, P_tac_-M), some strong promoter-5′-UTR (PUTR) sequences of *C. glutamicum* are also known, such as P_sod_UTR [27], P_tuf_UTR [28], but they are not sufficient. In a previous study, through different combinations of − 10 consensus and − 35 motifs, Rytter et al. constructed a synthetic PUTR library to modulate the gene expression in *C. glutamicum* [29]. Furthermore, by combining green fluorescent protein with fluorescent activated cell sorting (FACS), six PUTRs were obtained from the fully synthesized PUTR library [30]. Although native PUTRs and their sequences can be easily obtained through metabolic engineering of *C. glutamicum*, only few of them have been identified and characterized, making it difficult for their use in the rational design of complicated metabolic engineering strategies [31].

In this study, *C. glutamicum* ATCC 13032, the most widely used strain of *C. glutamicum*, was chosen as the host to perform screening of native PUTRs. A total of 90 PUTR sequences with different Transcripts Per Million (TPM) were selected based on RNA-Seq data. These 90 PUTRs exhibited a span of expression levels, and 16 strong PUTRs and a strongest PUTR were selected based on the strong P_sod_UTR in stationary phase. Because we are interested in using *C. glutamicum* for enhanced production of l-methionine and l-cysteine, nine common typical chemicals related to the biosynthesis pathways of these two amino acids were added to the culture medium to explore their potential effects on these PUTRs. As an important synthetic biology component, these native PUTRs with varying strengths could be useful in fine-tuning regulation of metabolic networks for microbial biosynthesis of useful products.

## Materials and methods

### Strains and plasmids

*E. coli* JM109 was used for plasmid amplification. *C. glutamicum* ATCC 13032 was employed to express fluorescent protein and measure PUTR strength. Plasmid pEC-XK99E was used for the expression of fluorescent protein with different PUTRs.

### Culture conditions

*E. coli* strains were grown in LB medium (10 g/L yeast extract, 5 g/L peptone, and 10 g/L NaCl) at 37 °C. *C. glutamicum* strains were cultured in LBB medium (10 g/L yeast extract, 5 g/L peptone, 10 g/L NaCl, and 18.5 g/L brain heart infusion) at 30 °C. For preparation of competent cells, LBB medium containing 91 g/L sorbitol and Epo medium (10 g/L yeast extract, 5 g/L peptone, 10 g/L NaCl, 18.5 g/L brain heart infusion, 30 g/L glycine, and 1 g/L Tween 80) were employed. The CGXII medium contained 5 g/L urea, 20 g/L (NH_4_)_2_SO_4_, 1 g/L KH_2_PO_4_, 1 g/L K_2_HPO_4_, 0.25 g/L MgSO_4_·7H_2_O, 42 g/L MOPS, 0.01 g/L CaCl_2_, 0.01 g/L MnSO_4_, 0.02 mg/L sodium citrate, 0.01 g/L FeSO_4_·7H_2_O, 0.01 g/L ZnSO_4_·7H_2_O, 0.2 mg/L CuSO_4_·5H_2_O, 0.02 mg/L NiCl_2_·6H_2_O, and 25 µg/L biotin. The seed medium was CGXII contained 40 g/L glucose and 0.5 g/L yeast extract. The fermentation medium was CGXII contained 40 g/L glucose. Typical chemical (5 g/L), including l-methionine, sodium thiosulfate, L-homoserine, l-serine, l-glycine, l-lysine, l-aspartic acid, l-alanine, and l-glutamic acid, were added as required. Kanamycin was added to the medium at a concentration of 50 μg/mL for *E. coli* and 15 μg/mL for *C. glutamicum* as required.

### RNA-Seq

RNA-Seq was performed by Sangon Biotech (Sangon Biotech, Shanghai, China) based on the HiSeq 2500 platform. Each sample was analyzed in duplicate. *C. glutamicum* strains were precultured in CGXII medium supplemented with 0.5 g/L yeast extract and 40 g/L glucose for 20 h in shake flasks. Then, 1% of these cultures were inoculated into fresh CGXII medium supplemented with 40 g/L glucose. l-methionine (15 g/L) was added as required. The cells were harvested after 15 h and washed twice with PBS. The total RNA was extracted by RNAprep Pure Cell/Bacteria Kit (Tiangen, Beijing, China), and stored in liquid nitrogen. Each sample was quantified by NanoDrop (Thermo Fisher Scientific, Waltham, MA, USA) and 2% agarose gel electrophoresis. All the experiments were performed in triplicate.

### Genetic operations

**Fig. 1:**
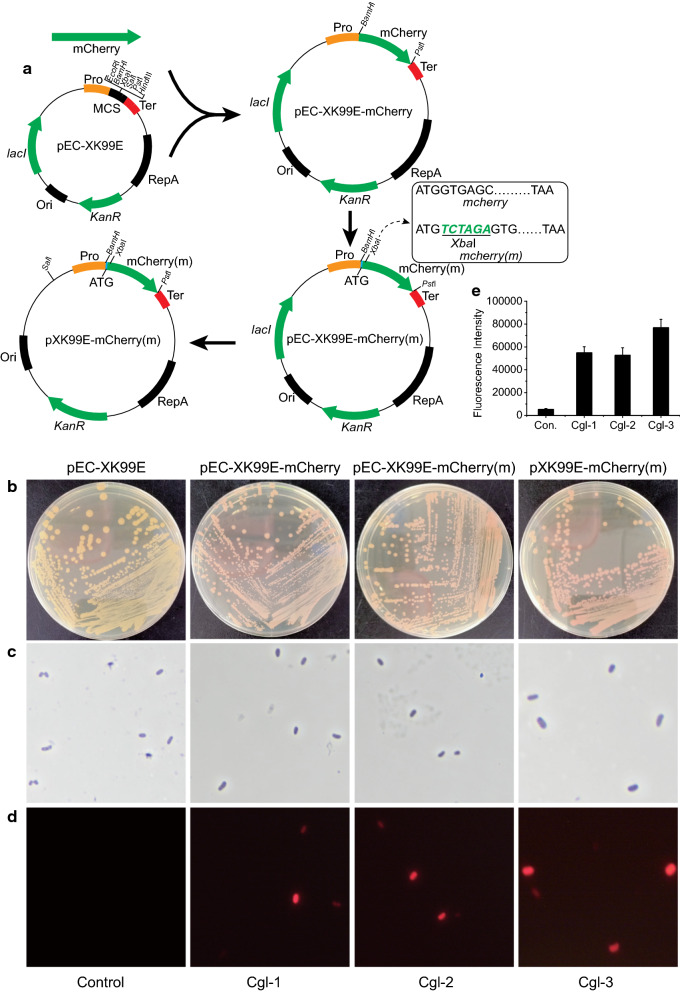
Construction of backbone plasmid. The strains were red on plates after 72 h of incubation. **a** Plasmids construction processes. **b** Growth of mutant strains on plates **c** Morphology of mutant strains under optical microscope. **d** Fluorescence of mutant strains under fluorescence microscope. **e** Fluorescence strength of backbone plasmid

All the PCR products were amplified by Phanta Max Master DNA Polymerase (Vazyme, China). The DNA was digested by FastDigest Restriction Enzymes (Thermo Fisher Scientific, USA), and plasmids were extracted by a SanPrep Column Plasmid Mini-Preps Kit (Sangon Biotech, China). The DNA fragments were purified by SanPrep Column DNA Gel Extraction Kit (Sangon Biotech, China), and ligated using One Step Cloning Kit (Vazyme, China). Primer Premier 5 software was used to design primers for the construction of plasmids carrying different PUTRs (Additional file 1: Table S1). The *mCherry* gene with suitable SD sequence (5′-AGAAGGACTAGTA-3′) was inserted between *Bam*HI and *Pst*I sites of pEC-XK99E, resulting in pEC-XK99E-mCherry. *Xba*I (TCTAGA), a restriction site, was inserted behind the initial codon ATG of *mCherry* gene, to obtain the plasmid pEC-XK99E-mCherry(m). The *lacI* gene was eliminated from the plasmid, while *Sal*I (GTCGAC) was inserted, resulting in the plasmid pXK99E-mCherry(m). Primers for construction of backbone plasmids listed in Table 1. Construction of backbone plasmid is shown in Fig. 1a.

**Table 1:** Primers used in this study

Primers	Sequence (5′ to 3′)	Restriction site
mCherry-F	agctcggtacccggggatccagaaggagactagta ATGGTGAGCAAGGGCGAGG	*BamH*I
mCherry-R	ccaagcttgcatgcctgcag TTACTTGTACAGCTCGTCCATGCC	*Pst*I
mCherry-XbaI-F	gtaatgtctagagtgagcaagggcgaggaggata	*Xba*I
mCherry-XbaI-R	tgctcactctagacattactagtctccttctggatcccc	*Xba*I
lacI-XC-F2	atttacgtgtcgacgcgcaacgcaattaatgtgagtta	*Sal*I
lacI-XC-R2	gttgcgcgtcgacacgtaaatgcatgccgcttcg	*Sal*I

**Fig. 2:**
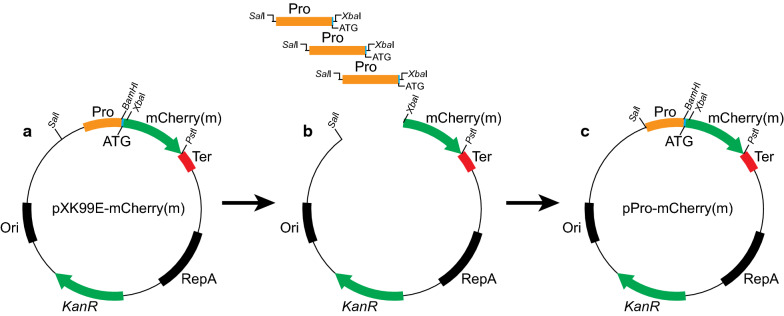
Construction of the PUTR library. With the backbone plasmid pXK99E-mCherry(m) as the starting plasmid, plasmids with different PUTRs were constructed. **a** Backbone plasmid; **b** Linearization of backbone plasmid by endonucleases (*Xba*I, *Sal*I). PUTR fragments were amplified from *C. glutamicum* ATCC 13032 genome; **c** Linking of linearized backbone plasmid to PUTR fragments

To prevent superfluous (or missing) bases from affecting the actual expression of the reporter gene, all the PUTR fragments were precisely amplified from the first base upstream of the initial codon ATG (or GTG), and seamlessly linked to the sites of *Sal*I and *Xba*I of the backbone plasmid. Finally, 90 plasmids harboring different PUTRs were successfully constructed and verified by Sanger sequencing (Fig. 2). Verfied plasmids were then transformed into *C. glutamicum* ATCC 13032, resulting in the PUTR library.

### Fluorescence and cell concentration measurements

*C. glutamicum* strains were precultured in CGXII medium supplemented with 0.5 g/L yeast extract and 40 g/L glucose for 20 h in shake flasks containing 25 mL of culture medium at 37 °C and 220 rpm orbital shaking. Then, 1% of these cultures were inoculated into CGXII medium supplemented with 40 g/L glucose in 48-well plates, with each well containing 2 mL of flesh culture medium, at 37 °C and 220 rpm orbital shaking. The cell fluorescence and cell density (OD_600_) were measured on a Synergy H1 Hybrid Multi-Mode Reader (BioTek Instruments, Winooski, VT, USA) at emission and excitation wavelengths of 587 and 610 nm, respectively. Fluorescence strength level is defined as the relative fluorescence unit divided by cell concentration (RFU/OD_600_) [32]. The *C. glutamicum* strains harboring P_sod_UTR was used as the control for strong PUTR screening.

## Results

### Obtaining of PUTR fragments based on RNA-Seq

To understand the transcriptional level of each gene and transcriptional intensity of each promoter in *C. glutamicum*, transcriptome profiling was conducted. Two groups of RNA-Seq data (with/without l-methionine) were obtained, in which 3136 genes were detected. In the test group, the highest transcript gene was *NCgl1929* (*CYL77_10110*), and its TPM, measuring the proportion of a transcript in the RNA pool, was 27617.38. There were many genes with lowest transcript, with TPM of 0.0001. A total of 90 PUTRs with TPM ranged from 2.33 to 23,149.19 were selected based on the RNA-Seq data. The selected PUTRs are divided into two types: (1) PUTRs of genes involved in l-methionine and l-cysteine biosynthesis; (2) PUTRs of some genes with gradient decrease in TPM. According to the physical distance between the genes in the genome and the normal length of prokaryotic gene PUTR, the length of the selected 90 PUTRs ranged about from 200 bp to 500 bp (Additional file 1: Table S2).

### Effects of backbone plasmid construction on fluorescence

To determine the strength of PUTRs, the *mCherry* gene was used as the reporter gene. The strain harboring the plasmid pEC-XK99E-mCherry, Cgl-1, showed obvious red color on the plate, indicating that the *mCherry* gene can be used as the reporter gene (Fig. 1b). When no restriction site is available, Polymerase Chain Reaction (PCR) must be used to linearize the vector for inserting PUTR fragments before the reporter gene sequence, but it would have the risk of gene mutation. To eliminate the risk of reporter gene mutation during plasmid construction, *Xba*I (TCTAGA) was inserted behind the initial codon ATG of *mCherry* gene, resulting in *mCherry(m)*. The strain harboring pEC-XK99E-mCherry(m), Cgl-2, also showed obvious red color on the plate, indicating that the *mCherry(m)* gene was feasible as a reporter gene (Fig. 1c). In order to facilitate the construction of subsequent plasmids and eliminate possible effects of LacI, *lacI* gene was eliminated. Cgl-3, the strain harboring pXK99E-mCherry(m), also showed obvious red color on the plate, revealing that the knockout of *lacI* gene was also feasible (Fig. 1d, e).

### PUTR strength under different growth phases

**Fig. 3:**
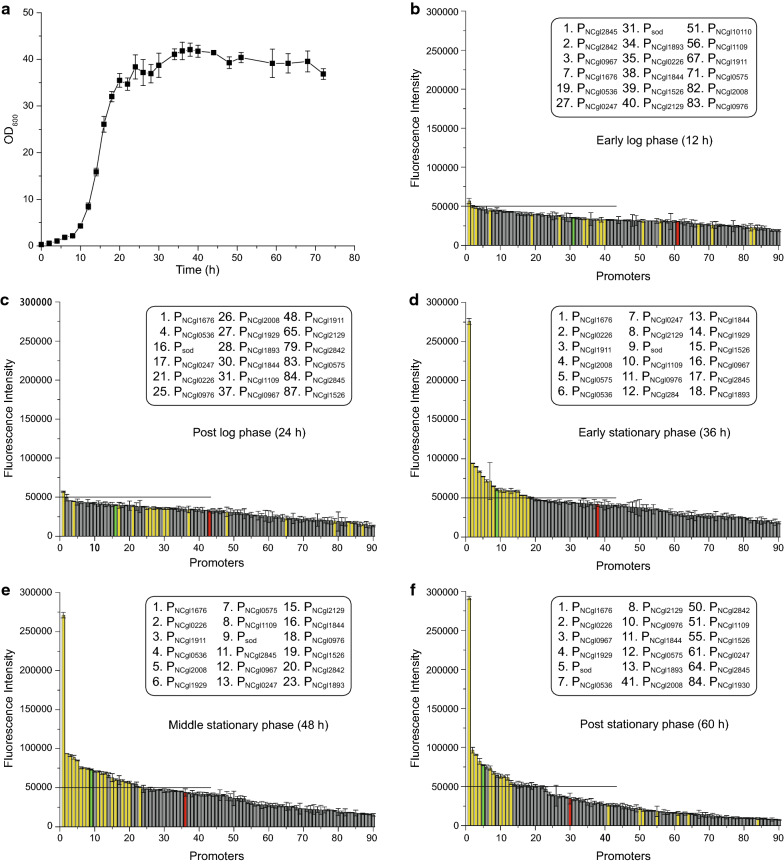
Fluorescence strength of 90 native PUTRs. In **b**–**f**, the line parallel to the X-axis is the dividing line of the strong and weak PUTRs. The green bar denotes the control PUTR (P_sod_UTR), yellow bars indicate the stronger PUTRs in the stationary phase, and the red bar represents the P_tac_UTR. The strong PUTRs are shown in the box, and the number denotes its position on the X-axis. **a** Growth curve of the strains; **b** Fluorescence levels in the early log phase (12 h); **c** Fluorescence levels in the post log phase (24 h); **d** Fluorescence levels in the early stationary phase (36 h); **e** Fluorescence levels in the middle stationary phase (48 h); **f** Fluorescence levels in the post stationary phase (60 h)

To quantitatively detect the expression strength of the PUTRs, the fluorescence levels of strains were measured at five different growth phases: early log phase (12 h), post log phase (24 h), early stationary phase (36 h), middle stationary phase (48 h), and post stationary phase (60 h) (Fig. 3a). When compared with the strength of the P_sod_UTR, the fluorescence levels of the strains in the log phase were generally low (RFU/OD_600_ = 50,000 as the control value) (Fig. 3b, c). In the stationary phase, the fluorescence levels of the strains became stronger and exhibited stable expression levels. During microbial fermentation to produce useful compounds, the biosynthesis of target products generally peaks in the stationary phase of cell growth. Therefore, a series of PUTRs with high expression strength were examined in the early and middle stationary phase of growth (Fig. 3d, e). In the early stationary phase of growth, the strength of the 17 PUTRs was as follows: P_NCgl1676_UTR > P_NCgl0226_UTR > P_NCgl1911_UTR > P_NCgl2008_UTR > P_NCgl0575_UTR > P_NCgl0536_UTR > P_NCgl0247_UTR > P_NCgl2129_UTR > P_sod_UTR > P_NCgl1109_UTR > P_NCgl0976_UTR > P_NCgl284_UTR > P_NCgl1844_UTR > P_NCgl1929_UTR > P_NCgl1526_UTR > P_NCgl0967_UTR > P_NCgl2845_UTR > P_NCgl1893_UTR. Most of these highly expressive PUTRs also exhibited stable expression levels in the post stationary phase (Fig. 3f). Among these PUTRs, P_NCgl1676_UTR exhibited more than five times higher strength than that of P_sod_UTR in the stationary phase, although its transcript proportion was not the highest based on the RNA-Seq data, indicating that its ‘RBS’ translation efficiency may be higher than that of the other PUTRs, or the short half-life of *NCgl1676* mRNA reduces the TPM value. Interestingly, the expression strength of P_tac_UTR (the common promoter P_tac_ combined with a common RBS (5′-AGAAGGAGACTAGTA-3′)) was not very strong, which resulted in the low expression strength of the plasmid pXMJ19 containing the combination of the promoter and the common RBS.

### Effects of typical chemicals on PUTR strength

**Fig. 4:**
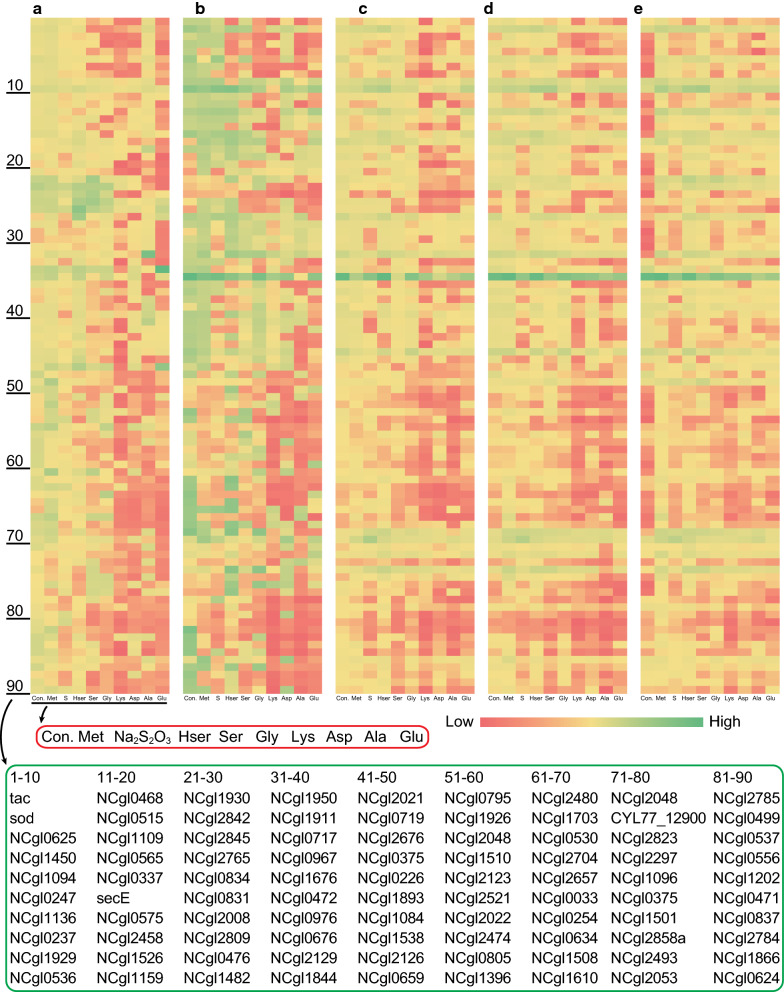
Effects of typical chemicals on promoter strength. The fluorescence strength of promoters in the medium with different additives at various culture stages. The X-axis denotes the additive components, which are listed in the red box in an enlarged manner. The Y-axis represents the genes corresponding to the promoters, which are listed in the green box in an enlarged manner. The five graphs represent different growth stages and their corresponding fluorescence levels (maximum (Green) and minimum (Red)). **a**: Early log phase (109307, 4374); **b**: Post log phase (56675, 4351); **c**: Early stationary phase (286247, 4796); **d**: Middle stationary phase (294678, 5560); **e**: Post stationary phase (300788, 7317)

*C. glutamicum* is a major strain used for the biosynthesis of amino acids. To facilitate the subsequent metabolic engineering of *C. glutamicum* to biosynthesize l-methionine, which we are interested in using *C. glutamicum* to achieve its fermentation production, nine typical chemicals related to biosynthesis of l-methionine were selected to study the response of these PUTRs to these chemicals. During the log phase of growth, the expression strength of some PUTRs was inhibited, while that of some of the PUTRs was activated (Fig. 4a, b). However, with the growth of the strains, the stress substances were metabolized, resulting in the decrease in the inhibitory effect and enhancement of fluorescence levels of the strains. In particular, in the stationary phase, the stress tended to become stable (Fig. 4d, e), which may be caused by the catabolism of the stress substances.

It has been reported that the protein BrnF encoded by the *brnF* gene (*NCgl0254*) is responsible for l-methionine and other branched-chain amino acids export [33]. In the present study, when the concentration of intracellular methionine increased, the expression of *brnFE* gene cluster was activated, and l-methionine was expelled from the cell. As shown in Fig. 4, with the addition of l-methionine to the culture medium (the control without l-methionine), the fluorescence level of P_brnF_UTR strain was 2–7 times stronger than that of the control strain in the early, middle and post stationary phase (39649.0/18466.8, 62903.1/17612.7, 76060.9/10741.6), indicating that double activation level presented a better effect (Additional file 2: Figure S1, S2). With this level as reference, the expression strengths of many PUTRs were activated or inhibited. For example, the fluorescence level of P_NCgl1202_UTR strain decreased by 90% in the log phase under l-lysine stress and began to recover slowly in the stationary phase, which may be because l-lysine was slowly metabolized and the inhibition was relieved. Taking P_brnF_UTR as the control, the inhibitory effect of P_NCgl1202_UTR by l-lysine is equivalent to the activation effect of P_brnF_UTR by l-methionine (Additional file 2: Figure S1, S2). Finally, we can use these two PUTRs as references to find out the PUTRs we want to be activated or inhibited by a certain typical chemical. Therefore, through the addition of different typical chemicals, the activation or inhibition effects of some PUTRs can be understood, and these results provide useful clues to engineer *C. glutamicum* for the biosynthesis of many useful compounds.

## Discussion

To address the needs to engineer precise expression of enzymes, in the present study, a total of 90 PUTRs with TPM ranged from 2.33 to 23,149.19 were selected based on RNA-Seq data. Then, the selected PUTR fragments, connected with *mCherry(m)* fragments, were inserted into the backbone plasmid, respectively. A PUTR library was constructed after the resulting plasmids were transformed into *C. glutamicum*. Subsequently, the fluorescence levels of the mutant strains were measured, and the effects of typical chemicals on the PUTRs were explored. Finally, 17 strong PUTRs were obtained, and some PUTR information on stress was also acquired. For example, the expression of P_brnF_UTR was activated by l-methionine, while that of P_NCgl1202_UTR was severely inhibited by l-lysine. These PUTRs could be helpful in fine-tuning the regulation of metabolic networks.

When compared with model microorganisms, such as *E. coli* and *Bacillus subtilis*, there are only relatively few synthetic biology tools available for *C. glutamicum*. Most of these tools involve the use of promoter or PUTR, and the limited number of available PUTRs restricts the improvement of these tools, resulting in the slow progress in fine-tuning the regulation metabolic networks in *C. glutamicum*. PUTRs, such as P_sod_UTR, P_tuf_UTR and P_H36_UTR [34], are the most commonly used strong PUTRs in *C. glutamicum*, and have been applied for the biosynthesis of many useful compounds [35, 36]; however, their expression strengths are not very strong [22]. More importantly, owing to the lack of efficient control elements, fine-tuning regulation of gene expression in multi-gene metabolic pathways is challenging. In comparison, our study acquired a strongest PUTRs, P_NCgl1676_UTR, with about fivefold higher strength than that of P_sod_UTR. For gene upregulation, plasmid-based expression will increase the burden of bacteria, and it will be more favorable if the strong promoter is used for genome integration expression [37, 38]. For multi-gene pathway, the heavy use of a same PUTR is likely to cause instability of plasmids or gene expression frames. Seventeen strong PUTRs with similar or higher expression level were obtained in our study can solve this issue, and can even be combined with other PUTRs to achieve fine-tuning gene regulation. Currently available prediction tools such as RBS calculator [39] and 5′-UTR designer [40] could be applied to rationally design 5′-UTR sequences with ideal strength to manipulate gene expression levels. Unfortunately, predicted sequence strength cannot always match with the actual strength [32]. These effects may be related to the secondary structures in the PUTRs, but further investigation is needed. In a word, by combining the existing vector expression systems [38, 41, 42] with gene editing systems [43–46], these PUTRs can improve the synthetic biology toolbox for *C. glutamicum*, as well as help in the development of more flexible applications for fine-tuning regulation of metabolic networks.

For construction and optimization of metabolic pathways, besides constitutive expression of genes, induction expression is often required. In previous promoter or PUTR screening studies, researchers tended to screen strong promoters or PUTRs; however, systematic studies on inducible promoters in *C. glutamicum* are limited. For instance, IPTG-induced promoters such as P_tac_ and P_trc_ have been widely used [47, 48]; however, IPTG is an expensive compound and is also not conducive to cell growth and industrial application. As the limited number of inducible promoters or PUTRs in *C. glutamicum* have been reported, a few amino acids, including l-methionine and l-cysteine, fail to achieve industrial fermentation through static or dynamic regulation of their metabolic pathways [49–52]. In our study, some PUTRs were strongly inhibited or induced in the early log phase, but tended to show the same level as that in the control in the late phase of growth, similar to that exhibited by P_iolT1_UTR [53]. If these mechanisms can be explained after further investigation, they can be engineered for designing dynamic regulatory systems to achieve more efficient biosynthesis of l-methionine, l-cysteine and their derivatives [51, 54, 55]. Besides, the substrates-inhibited or -inducible promoters or PUTRs can also be used in biosensor design [56–58], thus achieving rapid screening of high-yield strains with reporter genes. In conclusion, the results of this study provide a good toolbox for metabolic engineering of *C. glutamicum* for the synthesis of useful compounds.

## Conclusion

Based on the RNA-Seq data of *C. glutamicum* ATCC 13032 and the fluorescence measurements of the 90 mutant strains with the selected PUTR fragments, we obtained 16 strong native PUTRs and a strongest native PUTR. Furthermore, nine typical chemicals related to the biosynthesis of sulfur-containing amino acids were selected as stress substances to preliminarily explore the stress on these PUTRs. The expression of P_brnF_UTR was activated by l-methionine, while that of P_NCgl1202_UTR was severely inhibited by l-lysine. This study provides a good toolbox for metabolic engineering of *C. glutamicum* for the synthesis of useful compounds.

## Supplementary information

**Additional file 1: Table S1.** Primer sequences for construction of plasmids carrying different PUTRs. **Table S2.** The detailed promoter information.

**Additional file 2: Figure S1.** Effects of typical chemicals on promoter strength. **Figure S2.** The level of inhibition or activation of PUTRs by typical chemicals.**: > 1 represent activation; < 1 repersents inhibition.

## Data Availability

All data generated or analyzed during this study are included in this published article and its supplementary materials.
