# Supplementary material for: Obtaining a series of native gradient promoter-5′-UTR sequences in Corynebacterium glutamicum ATCC 13032
Source: Microb Cell Fact. 2020 Jun 3;19:120. doi: 10.1186/s12934-020-01376-3 (PMC7268698; doi:10.1186/s12934-020-01376-3)
Supplement: Supplementary file 1 — Additional file 1: Table S1. Primer sequences for construction of plasmids carrying different PUTRs. Table S2. The detailed promoter information. [file 12934_2020_1376_MOESM1_ESM.pdf]

Table S1 Primer sequences for construction of plasmids carrying different PUTRs.

Gene Label	Gene Name	Primer Sequence (5' to 3')
10110	NCgl1929	gcatgcatttacgtgtcgacCAGTGTTACCCCCTAAGACTACCCCT
		tcgcccttgctcactctagaCATGAGAATAGTCCTTCGGAAATAGTCG
02870	NCgl0536	gcatgcatttacgtgtcgacGCGTTTTCAGATCATGATTGATTGG
		tcgcccttgctcactctagaCATATCCTCCGCTTTTCCAGAACAC
02535	NCgl0468	gcatgcatttacgtgtcgacTTCACCGAAGACCGTAGGTCATCC
		tcgcccttgctcactctagaCATTACTTCGCCTCCTTCCTCTTATG
02765	NCgl0515	gcatgcatttacgtgtcgacTAGAGCACAACGGTGTTTTAAAGATCCA
		tcgcccttgctcactctagaCATTAGAGACGACCTGATGCCTTTCT

05845	NCgl1109	gcatgcatttacgtgtcgacATAGCGGACATTTTTTGACGCAGAT
		tcgcccttgctcactctagaCATTGTTCCTTTAAGTTACGCTAAATATCA
03015	NCgl0565	gcatgcatttacgtgtcgacCACGCTAAAATAGTGTGATTCTGTCCG
		tcgcccttgctcactctagaCATTCACAATCACTCCCCTTTTTTT
01835	NCgl0337	gcatgcatttacgtgtcgacTAAAATCTCCATAAGAGTAACGCCTGC
		tcgcccttgctcactctagaCATATTTTGCAGTCCTCTTGCAGATCA
02480	secE	gcatgcatttacgtgtcgacAATAAAACCAGATCAAGGGTGTTTTTTGTT
		tcgcccttgctcactctagaCATAGCACTCCTCTAAATCACTACTCATCC
03065	NCgl0575	gcatgcatttacgtgtcgacTTATGTGTCGAGGTGAATCTCCGGT
		tcgcccttgctcactctagaCATGTTCTCGCACCTTCCTGCAT

12840	NCgl2458	gcatgcatttacgtgtcgacCCGGTAGCTCCCTGACTACCTA
		tcgcccttgctcactctagaCATTTAGGTATTCCTTGGTAGGTAAACAAT
13090	NCgl2493	gcatgcatttacgtgtcgacAGCGAAATTTAGGCGATTTTTAAACG
		tcgcccttgctcactctagaCATTGCCTGACCCCCTTGGAG
02685	NCgl0499	gcatgcatttacgtgtcgacGGGGGTAGGGGTGTGGTTAGATATA
		tcgcccttgctcactctagaCATTACTGGTCTCCTGACCTGGATTAAT
02875	NCgl0537	gcatgcatttacgtgtcgacTAAGCTTTAAGCCTCCATATATCAGGGC
		tcgcccttgctcactctagaCATGTGGCAGTTACCTTTCCGGTT
02970	NCgl0556	gcatgcatttacgtgtcgacCTATGCAATTTCGGTTTAACCCAGTTTT
		tcgcccttgctcactctagaCATAATGACTCCTTGAATTAGTCGGTCT

06320	NCgl1202	gcatgcatttacgtgtcgacCTTGATCTGATCCGACAACCCAATG
		tcgcccttgctcactctagaCATATTAAACCCATCACAACACCCG
02550	NCgl0471	gcatgcatttacgtgtcgacAAATCTCCCCTTTACTTTCGCGC
		tcgcccttgctcactctagaCATGATGGGTCCTTCCAGCACC
04460	NCgl0837	gcatgcatttacgtgtcgacCCTCCGTGACTAGGCTAGATGACG
		tcgcccttgctcactctagaCATTTACTCTCGATTCCCTCAGGATTG
14580	NCgl2784	gcatgcatttacgtgtcgacACTCACAATTTATCTTGCCTAAGCGTAGT
		tcgcccttgctcactctagaCATACTCCCCACAGTACGTCCGAT
09795	NCgl1866	gcatgcatttacgtgtcgacGCCTGAGCGAGAAGTTCATCATGAC
		tcgcccttgctcactctagaCATGGCGATTAGTCTAACTCCCTTAACA

07960	NCgl1508	atgcatttacgtgtcgacAACGCTGAAGGAGTGCTGTCCAT
		ccttgctcactctagacatGTCGCTGATCCTAGCAGGTCATG
08465	NCgl1610	atgcatttacgtgtcgacTCGAAGTGCTCAAGATGGGTCG
		ccttgctcactctagacatATTCAACCTCTCATAGATGGGCG
07925	NCgl1501	atgcatttacgtgtcgacCGCAGACGCTATTCCAAACCTC
		ccttgctcactctagacatCTGTCACAATCTTTCAGGGTTGAAG
14990	NCgl2858a	atgcatttacgtgtcgacGGTCATTGAAGATCAGGAGGCC
		ccttgctcactctagacatTTCCCCCGTGACATCATTCTGTA
05780	NCgl1096	atgcatttacgtgtcgacAAAGGCTGATTTCACGACCAAGA
		ccttgctcactctagacatGAATGCCTCCTATACCCATTCCG

14770	NCgl2823	atgcatttacgtgtcgacCCCGTCTGACTACTTCCTCGCC
		ccttgctcactctagacatCGCGACAACACCGGTGTCA
02025	NCgl0375	atgcatttacgtgtcgacAGGAGGATTCGTCGAGAAGCG
		ccttgctcactctagacatGGGATCGCTCCTTCTGCGAGT
01400	brnF	atgcatttacgtgtcgacGCAACGGAGCTTTCAAAATTGTCT
		ccttgctcactctagacatCTCCAGGCTTGAATGAATCTCTTG
10705	NCgl2048	atgcatttacgtgtcgacACCATGTCCAACATTGCCAAGCT
		ccttgctcactctagacatCATTGAAAATCCAATCTAAAAGTCGAAA
tac	tac	gcatgcatttacgtgtcgacCACGAGGCGTTTTGAGCATG
		tcgcccttgctcactctagaCATTACTAGTCTCCTTCTGGATCCG

sod	NCgl2826	gcatgcatttacgtgtcgacTAGCTGCCAATTATTCCGGG
		tcgcccttgctcactctagaCATGGGTAAAAAATCCTTTCGTAGGTTT
metY	NCgl0625	gcatgcatttacgtgtcgacTTGTTAATTCTTAACAATGGAAAAGTAACA
		tcgcccttgctcactctagaCATTTGGAGGTCCTTAAGAGTTTTTAT
metH	NCgl1450	gcatgcatttacgtgtcgacGCGATGTGGGTGTGAGTCCAAG
		tcgcccttgctcactctagaCATTGTGTAGACAGCTTAGTACGGATTAGA
metE	NCgl1094	gcatgcatttacgtgtcgacTGAAAACGCAATCGGTTCACTTT
		tcgcccttgctcactctagaCATGTGAGTTAAAGACTTTCTGCTTGAG
lysc	NCgl0247	gcatgcatttacgtgtcgacCTTGATTCAGGGTAGTTGACTAAAGAGTT
		tcgcccttgctcactctagaCATCTTTGTGCACCTTTCGATCTA

hom	NCgl1136	gcatgcatttacgtgtcgacTTGAGCGAAGCTCCAAAATGTC
		tcgcccttgctcactctagaCATGATTCTCCAAAAATAATCGCG
aspB	NCgl0237	gcatgcatttacgtgtcgacTTTAGAAACTCAAATCCGCTCGCA
		tcgcccttgctcactctagaCATAACTGCGTACCTCCGCATGTG
gapA	NCgl1526	gcatgcatttacgtgtcgacTTTTTGAATGTGTCTGTATGATTTTGCAT
		tcgcccttgctcactctagaCATGTTGTGTCTCCTCTAAAGATTGTAGG
atpB	NCgl1159	gcatgcatttacgtgtcgacCTATAACGCGGGTTGAACCGAGAA
		tcgcccttgctcactctagaCATGGACAGTGTTGTAACGCTCAGC
10115	NCgl1930	gcatgcatttacgtgtcgacGGGCTAAGAACATGTACGATCTAACCG
		tcgcccttgctcactctagaCATGATGAAAAGCCTTTCAGTAAAGCTA

14870	NCgl2842	gcatgcatttacgtgtcgacACCTACTCCTACGACCCCGAAGTC
		tcgcccttgctcactctagaCATGTCGGTCGACCTTTCATTGAT
14910	NCgl2845	gcatgcatttacgtgtcgacGGACTGTTGTGCGGGTGTGTAAATT
		tcgcccttgctcactctagaCATACCATAAGAGCATCAACATTTGTTTG
14485	NCgl2765	gcatgcatttacgtgtcgacTGTTAAGTGTTAATGGTGGGAAAACTGG
		tcgcccttgctcactctagaCATTACTTCTCCAGATTTTGTGTCATTCG
rpmB	NCgl0834	gcatgcatttacgtgtcgacATGAGAACCACCTCTTCGATGACCTT
		tcgcccttgctcactctagaCATAACTTACTTTCTCCTAGCCGCCC
04430	NCgl0831	gcatgcatttacgtgtcgacCGAAAACACGTTAAGATCGACAACAG
		tcgcccttgctcactctagaCATAACTTACTTTCTCCTAGCCGCCC

10505	NCgl2008	gcatgcatttacgtgtcgacCTCAACGCGATAGGTTTCAACCATAG
		tcgcccttgctcactctagaCATGCCCATAAGCCTAGTACGTCATT
madh	NCgl2297	gcatgcatttacgtgtcgacGTAACGAGAAAACGTGAATTAGAAACGG
		tcgcccttgctcactctagaCATTAAACAATCCTCAATCCTTGTAGGG
14705	NCgl2809	gcatgcatttacgtgtcgacGTTGTCGAACTAGAAATCACCGACCA
		tcgcccttgctcactctagaCATTTGAGCTCCTTAAGCTACAAACACTC
02575	NCgl0476	gcatgcatttacgtgtcgacTGTTTGCATTTGATGAGGTTGTCCG
		tcgcccttgctcactctagaCATGAACCATCCTTATTTCGGAGTTGT
00200	NCgl0033	gcatgcatttacgtgtcgacGGGACAACGCATCATAAGTCTCAGAC
		tcgcccttgctcactctagaCATGCTTTTACTAATGTGGGGTCTTAGA

12900	CYL77_12900	gcatgcatttacgtgtcgacGAGATTATGTGGACAGTGAGGCGGAT
		tcgcccttgctcactctagaCATACCATAAGAGCATCAACATTGTTTGT
07820	NCgl1482	gcatgcatttacgtgtcgacTAGAAAGTCACATCACGCACGTACCC
		tcgcccttgctcactctagaCATTTCTAACTTTTTTAGGGTGAGATTTC
rpsB	NCgl1950	gcatgcatttacgtgtcgacAAGCATGGTGAGGGGTAATTTTAACTAC
		tcgcccttgctcactctagaCATGTTAAGCCCCTTTCTAAGTCTTAGG
10015	NCgl1911	gcatgcatttacgtgtcgacGCTTGAACCGGCATGAAAATCTC
		tcgcccttgctcactctagaCATCTGCTGCGTTCAATCCCTTT
03790	NCgl0717	gcatgcatttacgtgtcgacAACAATTCATCTAACGCCCACACGA
		tcgcccttgctcactctagaCATCGTGGTTCACCTCCGAAGTG

fumC	NCgl0967	gcatgcatttacgtgtcgacCAGTCTATATCGAACCGATGAGAGCAA
		tcgcccttgctcactctagaCATGAAATCACTCATTTCTTACTGAGAAT
08840	NCgl1676	gcatgcatttacgtgtcgacATGCGACAGTACTTTTCATTAAGCCTAAG
		tcgcccttgctcactctagaCATGTCCATAGTCCTAACCAATCATGTAA
03370	NCgl0634	gcatgcatttacgtgtcgacCGACTACTACTGGATTGCTGGCGA
		tcgcccttgctcactctagaCATGAGTCTCCTTGGTTGATGGGC
02555	NCgl0472	gcatgcatttacgtgtcgacCTCCAGTCCTTGTGCCTGAACGT
		tcgcccttgctcactctagaCATGTAAAACTCCCTTTCCCCCC
glpX	NCgl0976	gcatgcatttacgtgtcgacCCCCCTTTTGGGTGTCCAGA
		tcgcccttgctcactctagaCATCTGAAGGGCCTCCTGGG

03580	NCgl0676	gcatgcatttacgtgtcgacTAAAGAAATACCAATGAAAATTGGCAACT
		tcgcccttgctcactctagaCATGGTTTCTTAGTCTAAATCCTGAAGGG
11110	NCgl2129	gcatgcatttacgtgtcgacCTACACTTCTGGAGCGTTACGGTGC
		tcgcccttgctcactctagaCATGTACTTAACAATAGGCGATTGTGAGG
09685	NCgl1844	gcatgcatttacgtgtcgacATCACCGTGGAACAAGGACGATTC
		tcgcccttgctcactctagaCATAACTGGCCTCCTAAATTCGCG
hisD	NCgl2021	gcatgcatttacgtgtcgacCAGTATCTCAAAGGTGAAAGCGGGT
		tcgcccttgctcactctagaCATGGCGAAAAGTTCTCCCTTAAAA
10730	NCgl2053	gcatgcatttacgtgtcgacCCGTTGGCGAAAAATGTAGTGTG
		tcgcccttgctcactctagaCATGCCTTTCTCGACGTTGTCCT

NCgl0719	NCgl0719	gcatgcatttacgtgtcgacCCATCATGTTTGGCGCTCCATAT
		tcgcccttgctcactctagaCATGTCCTGGCGAGCTCCTTTTA
14040	NCgl2676	gcatgcatttacgtgtcgacCCGAGCGATCTTTATGGCACG
		tcgcccttgctcactctagaCATGGATTTCCCAGCCCTTCC
gnd	NCgl1396	gcatgcatttacgtgtcgacTGAGTGCTTCAAGTTCGTCTGTGGTT
		tcgcccttgctcactctagaCATGTTATTGATCGTACTTGACGGCA
02025	NCgl0375	gcatgcatttacgtgtcgacTCGAACATCCATTTTTGTGCCG
		tcgcccttgctcactctagaCATGGGATCGCTCCTTCTGCG
01240	NCgl0226	gcatgcatttacgtgtcgacTGGATCTGTATTTGACGTGGTTCGAG
		tcgcccttgctcactctagaCATGTGGTGCTCATCTCCTACTTCTAGTT

09925	NCgl1893	gcatgcatttacgtgtcgacGCGTGGTGCTTCTGTGAATAGAGTTG
		tcgcccttgctcactctagaCATCGATGTTGTAAACGCAGCAGTC
14585	NCgl2785	gcatgcatttacgtgtcgacGGAACTAGCAGTGGCCTTTTCAAAA
		tcgcccttgctcactctagaCATAGGGGGTACTTTCCCAAAGTTATG
fmt	NCgl1538	gcatgcatttacgtgtcgacGAGGAAGAATGATTTGCCGTTGC
		tcgcccttgctcactctagaCATGGTTGTGGGAAGGCTCCTTC
05720	NCgl1084	gcatgcatttacgtgtcgacGCCGACCGTATTCTTGTTGTTGAA
		tcgcccttgctcactctagaCATGGCAGGTACTCGCCTCTTTTC
13025	NCgl2480	gcatgcatttacgtgtcgacTCACAGATAGTATTCGGGCATTTCCT
		tcgcccttgctcactctagaCATTGAATACCTCCTCAGGTAATCGG

aceF	NCgl2126	gcatgcatttacgtgtcgacAAGCCTATTGTAGGGGGCATCTGTT
		tcgcccttgctcactctagaCATTATTTTAAGACTCCTCGCAAGTCGT
gltA	NCgl0795	gcatgcatttacgtgtcgacGTGGGTGGTCGGGAATGATGTAAC
		tcgcccttgctcactctagaCATATTTGTTCGGAAAAAAACTCTTCCG
10095	NCgl1926	gcatgcatttacgtgtcgacATATCCCGTGCTTGTTTATTCAGCTC
		tcgcccttgctcactctagaCATGTTCAACTTCCTTTTATCTCCGC
10705	NCgl2048	gcatgcatttacgtgtcgacATCACACTGGGATTACCCCGTGTA
		tcgcccttgctcactctagaCATTGAAAATCCAATCTAAAAGTCGAAAG
07970	NCgl1510	gcatgcatttacgtgtcgacCACACCAATTACTCATTCTGCGATCC
		tcgcccttgctcactctagaCATGACTGAAAGACAAGGTTGGGAAA

11080	NCgl2123	gcatgcatttacgtgtcgacGCATCAATCTTCTTTGGCAACTTCTTTT
		tcgcccttgctcactctagaCATGGATTTTAAGGTACACCTGCTACCT
13230	NCgl2521	gcatgcatttacgtgtcgacGCCCACGATGTTTAAATAGGCGAT
		tcgcccttgctcactctagaCATCTGAACTCCTCAACGTTATGGCTAT
10575	NCgl2022	gcatgcatttacgtgtcgacAAGTTCTCCCTTAAAAGGTTGAATCGAA
		tcgcccttgctcactctagaCATTCCTTCCAGTATCTCAAAGGTGAAA
cysE	NCgl2474	gcatgcatttacgtgtcgacGTTAACCACTCAAGCTCTTTGCTTGG
		tcgcccttgctcactctagaCATTTTTATTGTCGAGAGCATGCAGG
04295	NCgl0805	gcatgcatttacgtgtcgacTTTCTTCTCCTAAAAAGTGACACGGG
		tcgcccttgctcactctagaCATGAAAACTCCTAACAGTATGGGGTGTA

08980	NCgl1703	gcatgcatttacgtgtcgacTTGTTGACCACTGACAACTAGCAAGAA
		tcgcccttgctcactctaga CATAATAGTGGGTTTTGTACTCATGAAAA
14180	NCgl2704	gcatgcatttacgtgtcgacTGCTGATGAACTCTGAAACCGTCTC
		tcgcccttgctcactctagaCATGGTGACCAAGCATAGAACGACC
eutD	NCgl2657	gcatgcatttacgtgtcgacGGGGGTGAAGAGCTGTAAAGTACCG
		tcgcccttgctcactctagaCATACATCGCCTTTCTAATTTCAGCCT
02840	NCgl0530	gcatgcatttacgtgtcgacCCATCTGCATAAACTAAAACTGATTCCA
		tcgcccttgctcactctagaCATTGTCATCTCCTGACACTTGTGTGTG
metX	NCgl0624	gcatgcatttacgtgtcgacCTTTAAATAGACTCACCCCAGTGCTTAAA
		tcgcccttgctcactctagaCATGAAAATAACCGCCTGTTCTGG

pyc	NCgl0659	gcatgcatttacgtgtcgacCACTCCTGGGTTTTCACTTTGTTAA<br>tcgccccttgctcactctagaCATTAGAGTAATTATTCCTTTCAACAAGAGACC

Table S2 The detailed promoter information.

Gene Label	Gene Name	DNA Sequence (5' to 3')
10110	NCgl1929	cagtgttaccccctaagactaccccctttccattgcatacaaaggaaatacatatagacttttgggcattagattacctcgataaaagtttagggaatctaaattcattgatcaagacttgctgtcgcctagctctaattcacttgagcccggctgctaaaggtcaagatcattgaatgcactacttgctagcagtcatctgaaaaaacgacgttggttcgtagtcgctggaaatttaataattcctccgtccccttcaactagggggtggaaacccgactatttccgaaggactattctc
02870	NCgl0536	gcgttttcagatcatgattgattgggcgtgcctgcttttgtgtttttttagggaccccaatgcgcgtgattcaactcatgtttgatatgtgctcctaaggtgtgtaacctatatcgatggtgtgcgtacatcttgagtgacgcaaccattttgaagtggaaaaacttaaggcctcccgcaggggagtgttctggaaaagcggaggat
02535	NCgl0468	ttcaccgaagaccgtaggtcatccgcatgacggatcgaaggtttcctacccttaggaacggcccacgcaggagacacttgaacgccttagattccttatgtggaatgtatagcgccctgtgctcttgcacggggcttttctcattggtttattgaccgtgtgaaagctccgcggatcagtagattacacataagaggaaggaggcgaagta

02765	NCgl0515	tagagcacaacggtgttttaaagatccatgtaaacgtaaacacctactttgttgtaggccccccgccatgaatggcgatccgtgtgggaaatggtagcgagcaaaccgactattgagttcggtgagcg ttgagtggccagaaaacacacggttaacgtccaaggtgggaagggaaccccaacgagaaaggcatcaggtcgtctcta
05845	NCgl1109	atagcggacattttttgacgcagatcaccttactctgaaggataaggattcttagtgtcggtgcacttttactgatgtttcactgtggaggtcaacgactcaaagtcgagaattggtggcgcgtgtcactg gaattgacgcgtgaatggggtggaagtggacgtcgaaaagcatttttgagacgtttatgtgagcaatgtcccattttccctgctcacctgtatgggcacccgcggcggaagtggaattgcatatggag ttttgatgatatttagcgtaacttaaaggaaca
03015	NCgl0565	cacgctaaaatagtgtgattctgtccgaatctgttgttttagttttgaaactgcgggatcatggaaagtagtgaaaagtgaattttagttctgtgctttctctgccctttaagtgaaccttttgttggatcttgcat tgaaaaaatgaaaacctcgtcgggaatgcaacttgggatcacgtctcgggcaagaaacgtccttaaaaaaaggggagtgattgtga
01835	NCgl0337	taaaatctccataagagtaacgcctgctatgtctcttaaatttcatgtcctgccccacaaatttggaaattgcgctgaactaaaatcaatttcaagctaaattaaggaacctgagcttctgacctattcagcg ctggattttttctggcattttcttggcagtcagtgagttgctaacgttctattggttaaaaattgatctgcaagaggactgcaaaat
02480	secE	aataaaaccagatcaagggtgtttttttgtttcatcaagggatctttcacctgtagcaggtatgtcctaataaatattgcgagggttcgcgggattaatgtactctcgaaggttgaacacagggctgcgattg tgctggatcaaatgtctgcacgaaaaattgttatcgcccctggatgagtagtgatttagaggagtgct
03065	NCgl0575	ttatgtgtcgaggtgaatctccggtgaattcttatagataacttgtttttgcaggtcaggacggggttaaggggatgggtgttatctgtcagtatgtgaggagatcaaggtgttgggggttctagttgctaa gatggtgaaaacccgtgaggccaaaatccaactgggtgaattacccctgcataaatgcatgagggctttatacttgtcttattattaaacttttagggttttgatgcaggaaggtgcgagaac

12840	NCgl2458	ccggtagctccctgactacctaagccgagctttcggcgtgagatgacaacaattcgtggaacaaccagacgacgacgtgatctggggcttactcctacccgaaaactccagacccgcccagaacc ggaatcaggacatcaccgagggcacgtcggtggatccaggcttattcacaagcactcatgattttccaggggtaagatcaggcagttcccattgtttacctaccaaggaatacctaa
13090	NCgl2493	agcgaaatttaggcgatttttaaacgatacgaaaatcgcctactttggcattttattttcatgaacacgcaggtcaagggttagttgcagaaattttccgaataacaggctattgtgtctatcaggaatacag ttaatacatcttgaaaagcccatgggccatccgaattcccaggatcggcccgctcactccaagggggtcaggca
02685	NCgl0499	gggggtaggggtgtggttagatatacaggttgtctgtgtaggtcggactcgcggtgcttttccgagtcgcgtttccgagacccctcgacgaggccgtaaaacgccaagcgttcgttgcccacattcgt ggagtatgtagacatctgtagtacaaagaccacgcgtgtttgggacggaaatccagcacgcattaatccaggtcaggagaccagta
02875	NCgl0537	taagctttaagcctccatatatcagggccagtcggggatatcccggcaggctgacacaccttcaggcaacgatggctgaagccttgcgaatcacaatccacctcccggtttccgtccggggaaagta ttgcttggcaggggcgattatggggaaaaccatcgcaacaaccggaaaggtaactgccac
02970	NCgl0556	ctatgcaatttcggtttaacccagtttttcaaagaaggtcactagcttttccgctggtcaccttctttttggtttttcaacgcagagatagtacactttactctttgtgtgtggagtcaaacctcccctttaaggg gtgcgcttggacagcaggacaaattcgggtcaccaccggccgccgaatttagcttccttccgaacatattcctggctggcagttctagaccgactaattcaaggagtcatt
06320	NCgl1202	cttgatctgatccgacaacccaatgggggcaaaaatgtgtccgaccaaaaattgtgcagcacaccacatgcccgctcggacaatgtcgatttgttaatgaaactgcagctctggcgattaaataagat ggtcagagacagtttttggcctgtcaacccctgtgattctcttatttttgggtgattgttccggcgcgggtgttgtgatgggtttaat

02550	NCgl0471	aaatctccccttttactttcgcgcgcgattggtatactctgagtcgttgcgttggaattcgtgactctttttcgttcctgtagcgccaagaccttgatcaaggtggtttaaaaaaaccgatttgacaaggtcattcagtgctatctggagtcgttcagggggatcgggttcctcagcagaccaattgctcaaaaataccagcggtgttgatctgcacttaatggccttgaccagccaggtgcaattacccgcgtgaggtgctggaaggacccatc
04460	NCgl0837	cctccgtgactaggctagatgacggatccatcatataactcgcgggagattttggcctgggctagacatattccataatcaatttttagttacagcgcaacaggtgtaaggtattggaggttcattgccaaatcatggtgatgcatacttaccgaacccaactcgggcacgatctggcatccgctggaaccgacctgaagtttcaatcctgagggaatcgagagtaa
14580	NCgl2784	actcacaatttatcttgcctaagcgtagtatccggaccccgcattcacagaacttacgcgccgtatccgccatggaagatcctggcaattgtatgagattgtctgtttaaccgggctgtctttaaaagattttgcttttcgacgcaccgttcggccgttatctagatctggtcacaatgtcggtttttcatggattcatgtcctttatcggacgtactgtggggagt
09795	NCgl1866	gcctgagcgagaagttcatcatgacttaagtttttcttttcatccattgtttatctattgtttcacgtctgctgtcgccaaggctaggtgtcgggcaacgccgtgagcgatcaagccaccataaacacctaaattgagcgctctattccatcaacgcgggggcaatttctcacgtgtaggaaaagtgactattgcctgttaagggagttagactaatcgcc
07960	NCgl1508	aacgctgaaggagtgctgtccatcatcccatccgttgcgctgtcccaaggtatggttgacgcattcaacggcgaacttccgtggctccagctaggaattttggtgggctggctaattatcaccggcgtggccgcaaacaagctatttaacttctctgcgagccgctagatatacccttagtcggaaaatatccttaaaagttcccgggagtttcagtggcaaataccaccacttccataccgggaacaattgtataaaactagccatgacctgctaggatcagcgac

08465	NCgl1610	tcgaagtgctcaagatgggtcgatatatggattgaggtcattcaggcggctgggcgcgattttaggggccctcacttttggggccggtggggcagttgtgaatcctgaaagcttccagggcaaggatccaccacaaaccaggctggactctagaaatcggtcctaaagttgcgcacgcaggcagataaagcggaaatatccaccacagccagcaggaaatccgctttttgtggtgaatattcgcacattttttctattcgtgtatgggtgcatgtggtaatgccttcccgcccttgagttctaccccgaaacctgcgcaagacacccccagaatgggaacgtagtctagatcacacgacttcgaaaagtggctccaagctggatagactctatttacctactggtaacctccgcttgatatttccctctggggaactaagtgggggaagatttcgacaactaaccgggcgcaaagatgaaactaatgcgtccgaccacggcgaaaaggaagtttcgcccatctatgagaggttgaat
07925	NCgl1501	cgcagacgctattccaaacctcgagattgaaaccggccagatcgttggcgcaggacacgcagcaaccgtcggtcgtttcgacgacgagcacgtgttctacctccaggcccgtggtattcctgcagaggaagcacgccgcctcatcgtccgcggtttcttcaacgaagtgatcaacaaggtcccagttgaatccatccgcggggaattggacaaccgagtcagctcggaactcgcagttcttggcatgtaattaagccaaagtaactaagccaagtggctaagcacagttacttggccaagctgggcggcagaaaaaccggcccagctaatacttcagtttaaaattcgcttcaaccctgaaagattgtgacag
14990	NCgl2858a	ggtcattgaagatcaggaggccgagatcgccgagatggagcagatgctcaacgagctctgaacaaggacattccccgaccaccccagatgagaaacacgaccatacgaccatcgggccaaaccaggatttccgggaaggacaatcagcatgccgaataaaatcaaggttgcagtaaacgggtacggtgtcatcggtaaacgtgtagccgacgcggtgagagcccaggaggacatggaactcctcggggtcagcgacgtcaccaccgattaccgatgacaccgtacccggatgtaggtcttatccacccgccaggaactggcctgctagtcaggtacctgccggtaccaccgtgtttgcttgtccagctcaggggcgtatgtctggacccagcggtgagaatcgtggtgtgatcgaccggcacgccccgctgaagtcatcgtttcctccagatcttaggtcagctcaccccgtagcggcagtgacctgcgcactgcctacagaatgatgtcacgggggaa

05780	NCgl1096	aaaggctgatttcacgaccaagatccccaaaagttcgacctctcagaatcgcttataagggcctttcgtggccccgtccatacaaacgcacattcggaaaaatgaagcccttaaatcgccatacagcacctctctcgaaatgcgcaaccacggcaattcagccaatctgcaataaaaaagtaaaacttatttagcggcccgtgaagatccccgaaaggtcacaatgaccagccattttgcctaaaccgtgtgctggattaaataggtgttaaatattaaaaactagcgagccctgaattaatctgttaaggtcctacatggacttcagttagttgaggctgaagtttttgacatgtatgtctcgtccgagttcgccgccatccaggtggtgcaccgaggtggattcatggtgaatccttaatcgagtgaggccccagtccagggaagcccgccgcattaagtgagttcctgatggcttcgggtggaggatgtagaactttttgtacatggtctgtggcacgcattgtttcgtttgccctgccatgttggtgttttaagtgaaggccgccttttgcggaatgggtataggaggcattc
14770	NCgl2823	cccgtctgactacttcctcgccgaggattaccaccagcaatacctggacaagaatcccgatggctactgccctcatcactccacgggcatcccgtgcggggtagaagcttaaaagatttttgcttttcgacgcatccctccacctcggattacgcctgaggcatgactatcgtcgcgcttctttccgacctggacaacacgcgtagctaccgcgaagaactgtactagttcttccatcagcacccgcagttgtctttgcaagagttcgaaacatccaacgcattggtgacaccggtgttgtcgcg
02025	NCgl0375	aggaggattcgtcgagaagcgcgcgccaggtttccacaggaaggctggattgatgaggaaatcacacgcaataatcgaacatccatttttgtgccgctaatttggttcctgatcgcggcgattagtttcgcgatatacaggttcgtcgacgccacatggggctcgattatcgcggcagtaattgcaatgaccctcacgtggtttacgccaggcatgttcccgcgaagggttgacccatacccctagggggtatacagtgagtcatgtaaacatactcgcagaaggagcgatccc
01400	NCgl0254	ctccaggcttgaatgaatctcttgcgtttttttgcacactacaatcatcacacaattgccgggtagttttgttgccagtttgcgcacctcaactaggctattgtgcaatatatgaagctagattccattgatcgcgcaattattgcggagcttagcgcgaatgcgcgcatctcaaatctcgcactggctgacaaggtgcatctcactccgggaccttgcttgaggagggtgcagcgtttggaagccgaaggaatcattttgggctacagcgcggacattcaccctgcggtgatgaatcgtggatttgaggtgaccgtggatgtcactctcagcaacttcgaccgctccactgtagacaattttgaaagctccgttgc

10705	NCgl2048	accatgtccaacattgccaagctcaagcaggtgctggtggacaacaagggtgaatctgatgtgtacctcaatttgatcgatggggataactccacggtcatgattttgggtgatcacttaagagtcaaccgatccgcaagtttgatgggcgacctcaaggcaacgatggggccaggcatcctcggttaatcacatcacactgggattaccccgtgtaggggtgaaaacccgaatgatgaataaaattccgggtgcagtgaccgtaggtgaggtaaacgcggttagagtcgaatgagagtttgatactttctttcgacttttagattggattttca
tac	tac	cacgaggcgttttgagcatgccgcagatacccaaaattttggggaagaattaggcaggcatcagaagctggcgatgtggtgattttggacgtttgagctgttgacaattaatcatcggctcgtataatgtgtggaattgtgagcggataacaattgcggccgccttaaggggcccgttaacggatccagaaggagactagta
sod	NCgl2826	tagctgccaattattccgggcttgtgacccgctacccgataaataggtcggctgaaaaatttcgttgcaatatcaacaaaaaggcctatcattgggaggtgtcgcaccaagtacttttgcgaagcgccatctgacggattttcaaaagatgtatatgctcggtgcggaaacctacgaaaggattttttaccc
metY	NCgl0625	ttgttaattcttaacaatggaaaagtaacattgagagatgatttataccatcctacaccatttagagtggggctagtcataccccataaccctagctgtacgcaatcgatttcaaatcagttggaaaaagtcaagaaaattacccgagaataaatttataccacacagtctattgcaatagaccaagctgttcagtagggtgcatgggagaagaatttcctaataaaaactcttaaggacctccaa
metH	NCgl1450	gcgatgtgggtgtgagtccaagaggtggcttttacgtcgtcaagcaattttagccactcttcccacggctttccggtgccgttgaggatagcttcaggggacatgcctggtgttgagccttgcggagtggagtcagtcatgcgaccgagactagtggcgctttgcctgtgttgcttaggcggcgttgaaaatgaactacgaatgaaaagttcgggaattgtctaatccgtactaagctgtctacaca
metE	NCgl1094	tgaaaacgcaatcggttcacttttaatcctctccctggagccccggatgatgaggaacgccaaagctttctgaatggaaattttaagcgttaagtgggacgacctcgattacaaaaaggcgaggaaacccccggggcagctttctgccacccggtgatttcgcgaaccttgaaacatcgtcagaagattgccgtgcgtcctagccgggatccgcacgttcggctcaagcagaaagtctttaactcac

lysc	NCgl0247	cttgattcagggtagttgactaaagagttgctcgcgaagtagcacctgtcacttttgtctcaaatattaaatcgaatatcaatatatggtctgtttattggaacgcgtcccagtggctgagacgcatccgct aaagccccaggaaccctgtgcagaaagaaaacactcctctggctaggtagacacagtttataaaggtagagttgagcgggtaactgtcagcacgtagatcgaaaggtgcacaaag
hom	NCgl1136	ttgagcgaagctccaaaatgtcctccccgggttgatattagatttcataaatatactaaaaatcttgagagtttttccgttgaaaactaaaaagctgggaaggtgaatcgaatttcggggctttaaagcaaa aatgaacagcttggtctatagtggctaggtacccttttgttttggacacatgtagggtggccgaaacaaagtaataggacaacaacgctcgaccgcgattatttttggagaatc
aspB	NCgl0237	tttagaaactcaaatccgctcgcagttggcgttttctggggcggttcagctagagttatgcgaaggatcccgtgcggcgtttatcttgtgaactcccccagggcaggaatgcagcaagggtcagcga gctctgacgggtgcgcgggatcccctaaaacgtctagagtagtggcttgaggtcactgctctttttttgtgcccttttttggtccgtctattttgccaccacatgcggaggtacgcagtt
gapA	NCgl1526	tttttgaatgtgtctgtatgattttgcatctgctgcgaaatctttgtttccccgctaaagttgaggacaggttgacacggagttgactcgacgaattatccaatgtgagtaggtttggtgcgtgagttggaaa aattcgccatactcgcccttgggttctgtcagctcaagaattcttgagtgaccgatgctctgattgacctaactgcttgacacattgcatttcctacaatctttagaggagacacaac
atpB	NCgl1159	ctataacgcgggttgaaccgagaaacccctcaaggcagcagacaatagccgcaaggggttttgcggagcacgtcccctgtgatcgttgcgctgatgtgcgacggagtccgtagcgattacagcga gtttttcagacgtccatcgcaccgtgcacaacaacatttcaggtgcacggcccgaacacgggagagaacgctgagcgttacaacactgtcc
10115	NCgl1930	gggctaagaacatgtacgatctaaccgtcttgggtggcattgcttcccttctcggtgtcatcgtattccccgcttacaactacttggtttacaccggtgttatcaagggctaaatcgcctaacctctatcttc atctatagcttagctttactgaaaggcttttcatc
14870	NCgl2842	acctactcctacgaccccgaagtcaggttccgctccatagctgcacttggcacggcatggaattagtgtcaaaagcctcaaaaatactggtactaaccagctgtgcggatcgggtatccgcgctaca cttagaggtgttagagatcatgagtttccacgaactgtaacgcaggattcaccaatcaatgaaaggtcgaccgac

14910	NCgl2845	ggactgttgtgcgggtgtgtaaattaattccagtcagcgcgaccaacaacgccccaacacataagagattatgtggacagtgaggcggatctaggaaaacaaacgctcgacaaaccaacaacacttcatcgaagtcaacaaacaccgatttggtgaaagtaaacaagatgaagtaacgttgaacaagctgccaacaagacaccaacacaaacaaatgttgatgctcttatggt
14485	NCgl2765	tgttaagtgttaatggtgggaaaactgggaaagttgtcccctggaatgtgtgagaattgcccaaatctgaacccaatggccatggacggggaatgaactgtcggagaacggttgaggttaattcttgaaaccacccccaaaataggctatttaaacgggtgctctcatattaaagaaagtgtgtagatgcgtgtgggcagggggtaggtccactggtaatgacaaatgtgtccgttgtctcacctaaagttttaactagttctgtatctgaaagctacgctagggggcgagaactctgtcgaatgacacaaaatctggagaagta
rpmB	NCgl0834	atgagaaccacctcttcgatgaccttgccccagcggttgaacatgcccgaaaacacgttaagatcgacaacagttaaggcttccggggcgatctaaacccgcattgaataggcgattaggtgttttggggcaacgtatgtaaacttgtcccttgctgttgtcgtaaatattcgttatcgccccgtcagctggcatgctcgcgccccggtcacccggtgcggcgcgtcttctaactgaatgtgggcggctaggagaaagtaagtt
04430	NCgl0831	cgaaaacacgttaagatcgacaacagttaaggcttccggggcgatctaaacccgcattgaataggcgattaggtgttttggggcaacgtatgtaaacttgtcccttgctgttgtcgtaaatattcgttatcgccccgtcagctggcatgctcgcgccccggtcacccggtgcggcgcgtcttctaactgaatgtgggcggctaggagaaagtaagtt
10505	NCgl2008	ctcaacgcgataggtttcaaccataggcctgacctggctgagatgtttttggtagaaaaaccgagtgcccgaattgttttgtgggtgcccggttttttctgatttaagcacgtcagaggcgtagaacattgtctgttcacactctgggtcgcaagattcatcgagaattaatggtagtacctgtggcttgagggggaatgacgtactaggcttatgggc

madh	NCgl2297	gtaacgagaaaacgtgaattagaaacggggttaaggtaaatatcaaagataacaccatcggcaaatcccagctgacaactataaatggtgcccgatatcaggaaaaattgcttgcacaccgcgccgattccccatgatgccctaacatcttgcaggtgaggggtacatattggggcaattcgggggtaattttgcagtatcgtcaagatcacccaaaactggtggctgttctcttttaagcgggatagcatgggttcttagaggaccccctacaaggattgaggattgttta
14705	NCgl2809	gttgtcgaactagaaatcaccgaccacgagatggaacgcttcaagcattccgcaaataccctgcgcgaaattcagaagcagttcttctaaatctttggcgcctagttggcgacgcaagtgtttcattggaacacttgcgctgccaactttttggtttacgggcacaatgaaactgttggatggaatttagagtgtttgtagcttaaggagctcaa
02575	NCgl0476	tgtttgcatttgatgaggttgtccgtgacatgtttggtcgggccccaaaaagagccccctttttttgcgtgtctggacactttttcaaatccttcgccatcgacaagctcagccttcgtgttcgtcccccgggcgtcacgtcagcagttaaagaacaactccgaaataaggatggttc
00200	NCgl0033	gggacaacgcatcataagtctcagacaccttgcggtcgcggtaatccgcataagttccataagctttacgtgcgccatccactactaacttgaggttgccaatcgtgctcatattcaactctccttaaattggtcgcgttcttctaagaccccacattagtaaaagc
12900	CYL77_12900	gagattatgtggacagtgaggcggatctaggaaaacaaacgctcgacaaaccaacaacacttcatcgaagtcaacaaacaccgatttggtgaaagtaaacaagatgaagtaacgttgaacaagctgccaacaagacaccaacacaaacaatgttgatgctcttatggt
07820	NCgl1482	tagaaagtcacatcacgcacgtacccatttcgagcaaatccgacaaaaccgctgcctagggacattagacacgctagcaggccaaaattccatgacgttattgacgcgccaagaaccccaactttcccgccagaacgcttgtactgttaggataatgaagacgtagggtccttttccacagttctgtggaatgagaatccgatgtttttctcacgccggctcagccgaagcagacgccgtcgcgaaatctcaccctaaaaaagttagaa

rpsB	NCgl1950	aagcatggtgaggggtaattttaactactgactacgcgcggtggcgttgaatgcacattcaactccggagcaacgaggtcccaaacagctcattcgagttttagggtgttgacctgggaaaccaccgctagggtgcaggataaaaacctgcacaaattttcaaaaccgaaaccctaagacttagaaaggggcttaac
10015	NCgl1911	gcttgaaccggcatgaaaatctcgtaccggttttgggctcacaaggccatataggaactttgtaattagttgcaggttccaattttgggtcaatgtagcgtaatattgttcaaggcccatgtgcgggctgtggaggacgtgcattcacgttctggtcaaatgaaaaacggtgaaagggattgaacgcagcag
03790	NCgl0717	aacaattcatctaacgcccacacgattcagacagcacatcaacgaactttaaaatcgctaattacctgcatatttacttggcgacctattcaagaaagccaagacatgatagcgtttgttattgacttgttattttccagttttcagacaactactgcacttcggaggtgaaccacg
fumC	NCgl0967	cagtctatatcgaaccgatgagagcaatccccaagtatttcccacccctgtttttaggtacacctaccgccgaattttggacgttaaacaagcctgcacccccacatttaggagtggattgtgccttatttctcacactttctattacccacctcactctaggggtggactccagtgtttcgcgacaacacaatgagtaagcttgtgacagccgtatttaattctcagtaagaaatgagtgatttc
08840	NCgl1676	atgcgacagtacttttcattaagcctaagaaaattcctttaattgacacttaattgaccaataagagtcgattagattgcattattaggtaatctagtgatttaatggagaataagagcaactggtgaagaaaaggcttgatgaaagaagtttttttatctagctagatgttcaatcacgagctttaagaaagtatgtcaataactttgacataacctaaacacaataaattatgtagtattatgtgacactaagttattacatttattatatgattggttaggactatggacatg
03370	NCgl0634	cgactactactggattgctggcgaaagtggtgtcgtgaccagcattcgtcgatctctagtgaaagagaaaggcctcgaccgttcccaagtggcattcatggggtattggaaacacggcgtttccatgcggggctgaaactgccaccataggcgccagcaattagtagaacactgtattctaggtagctgaacaaaagagcccatcaaccaaggagactc

02555	NCgl0472	ctccagtccttgtgcctgaacgtggaggttctctccgcagacggcactccaatggagctcgcgggtgacgacgacgacttcgatcaggcaggcgcctcacttggcatcaacctgtcccgtgacgag cgttccgacgccgacaccgcatagcagatcagaaaacaaccgctagaaatcaagccatacatcccccggacattgaagagatgttctgggggaaagggagttttac
glpX	NCgl0976	ccccctttgggtgtccagaatccaaaattccgggcacaaaagtgcaacaatagatgacgtgcgggttgatacagcccaagcgccgatacatttataatgcgcctagatacgtgcaacccacgtaac caggtcagatcaagtgccccaggaggcccttcag
03580	NCgl0676	taaagaaataccaatgaaaattggcaactctttacacccaatctttaagacatggggggtggcgctgggctaatataaccggttagcgaaacgattagtcccttgttagggggattaaccctcgaagtg ggtcgtattttggcgtttgtatgttcacacaagaaccctgcacaacgccttcaaagtacgtcgaccacgaccaagcgcattattcactctcacccttcaggatttagactaagaaacc
11110	NCgl2129	ctacacttctggagcgttacggtgcttccgaagacaccccagtggtgtccttcaactaagcccgaagttttttaaccgccgcattcgatcaccaaatgtggcggttttgcgtcgaaaagcgtgctctttct acacctctttgaggttcattttcgcggtttcctcacaatcgcctattgttaagtac
09685	NCgl1844	atcaccgtggaacaaggacgattcggcgcaatgatgaaggtcacatcggttaacgaaggccccttcaccgttttggtcgagtgctagccagtcaatcctaagagcttgaaacgccccaatgtgggg gtgttaagaactccataaaagcgcttgggaactttttgtggaagcagtccgttgaacctcttgaaccgcgaatttaggaggccagtt
hisD	NCgl2021	cagtatctcaaaggtgaaagcgggtttaattcaggtaaatctggggtggtcattttaagttttaagtctaattcaaatgaactctgatgtacccaaatcagaaacttgttacgtggggaatacaataggtaa acatgcgggcttaagaacttgtgttgaggccgcttggattcgggcaccgagctcgaagaatttcgattcaaccttttaagggagaacttttcgcc
10730	NCgl2053	ccgttggcgaaaaatgtagtgtggccggaggggcagttagacgatatcgatgcggattaagacataaccattcgctaatctttcgacgccccttctgaggtggggatttcttttcatcccctttaattattt tcggaaatttatacagcaatcctcgaaatcctaataaagatcccttatcgtgggagaggtacggtagttcgttcgaggacaacgtcgagaaaggc

NCgl0719	NCgl0719	ccatcatgtttggcgctccatattctgcgggccaggtgctggagaattacccatttatccgcgaactcgtcggtgagggctctgaagctcttcgcggtgctgcagcgcaggttttggaagaggcagatgtggaatatgacctcgaagcttatttagaggccctcaactagccctccactaaacagcttcaatcaattcggtgtccactccaacatgtagagtggtgcgcgttaaaaaagttttcctaattttcattttcttaaaaggagctcgccaggac
14040	NCgl2676	ccgagcgatctttatggcacgcgtgtgctgcgtgatctcaatggtcagtccagtattttccaagattccaccgacgcggatgagccaccaaaaaagtggtagaaaactggtgtttttcggccgtgtccaccccaggttctatgctgtaacaaacgcgggtttaaacctcaatcatcaaattagggaagggctgggaaatcc
gnd	NCgl1396	tgagtgcttcaagttcgtctgtggttaaagctctggtggcggcaagttctgcaagcgaaagcagatcttggggttgatcatcgcgggaagtcataattaattactctagtcggcctaaaatggttggattttcacctcctgtgacctggtaaaatcgccactacccccaaatggtcacaccttttaggccgattttgctgacaccgggctatgccgtcaagtacgatcaataac
02025	NCgl0375	tcgaacatccatttttgtgccgctaatttggttcctgatcgcggcgattagtttcgcgatatacaggttcgtcgacgccacatggggctcgattatcgcggcagtaattgcaatgaccctcacgtggtttacgccaggcatgttcccgcgaagggttgacccatacccctagggggtatacagtgagtcatgtaaacatactcgcagaaggagcgatccc
01240	NCgl0226	tggatctgtatttgacgtggttcgagaccccgcggtgttgcacaaagtggaagtcagtggaggaatcctcgagcctgaatgtgctgccttgatgaccgaatttttcgaacttcacaggtaacggatttatatcaatttcagggcgtggcgagcttttagtgattcacgctcctacggtgggtatcacaaatacctcaactagaagtaggagatgagcaccac
09925	NCgl1893	gcgtggtgcttctgtgaatagagttgtttgagcgactagagttaaggccatgactgtcagaaacggcgcacccaagcggggaagctcacgatcatcgggaaagtcgacggggccaaaaaccaccgcgacgaccaggcgaagcaaaccaacaaccagcacctctcggggtggccggtcagagactgctgcgtttacaacatcg

14585	NCgl2785	ggaactagcagtggccttttcaaaagcttggagccaatcattggccaaggtggttaagtcttgttcgggcaaagcatcctcctttaaaaagtgtcgtgactcactttctactctataaagtgtttcaagtcat tgagggtgcccccgtttgtaattcttgcaaagtgggtattgtgcactttttgcgaggatctatcgaatgaattggcaaaacataactttgggaaagtaccccct
fmt	NCgl1538	gaggaagaatgatttgccgttgcgtattcagatggatccgattcacatcggataaaactgctggtgaaaggcctaatggggtcgtgtcccatgtgggatataagatggggagacaagatcatccacc cataggaattattgttgaaggagccttcccacaacc
05720	NCgl1084	gccgaccgtattcttgttgttgaacaaggacgtatcattgaggacggatctcacgacgcgttgttgtctgctaacggcacctacgcccgcatgtggcatttaatggcctgacacgttatttttaggagaa ctgtcaacaaattaatgctacaactggggcttaggcataatcagccaacgaccaacgttacagtggataaaacaaagctcaataaaccctcaagaagcaaggaaaagaggcgagtacctgcc
13025	NCgl2480	tcacagatagtattcgggcatttcctgtcacgatggtttatccttgggacacaacatcaaagtggggtacatcatatgcttccggttgaagtgacctatctgaaaagattggtcgaaccttgaagcaatgg tgtgaactgcgttaacgaattttgtcggacgttaaaatggtcgcattctgcttgctgaagtggcacacctatgtgttctgcttgggtatagcagtgcgggaaaaatttgaaaaagtccgattacctgagga ggtattca
aceF	NCgl2126	aagcctattgtagggggcatctgtttagcttgatatgacccgaacaccacacatcacaaattgaatcggtatcctttggggtattagtttccgttttaacgacacgacttgcgaggagtcttaaaata
gltA	NCgl0795	gtgggtggtcgggaatgatgtaaccaacgtgattgtgggggaattggctctcacttcggatatggctaaaccgcatttatcggtatagcgtgttaaccggaccagattgggaaagaaatgtgtcgagt aacaaaaactgacatgcgcttggcgcatcccagttggtaagaataaacgggactacttccgtaatccggaagagtttttttccgaacaaat
10095	NCgl1926	atatcccgtgcttgtttattcagctcgaggtggcaggcgtacactctatattcacggacaatgtgtacccacgctttcttgtaagaaacaagaagggtaacgccccacgcgtcagtcaaaaatatggcc aacacttgcattcgggtgctggcgatcatttatgagatgacgccttgtgttggtgttcggcagagaactcgcggagataaaaggaagttgaac

10705	NCgl2048	atcacactgggattaccccgtgtaggggtgaaaacccgaatgatgaataaaattccgggtgcagtgaccgtaggtgaggtaaacgcggttagagtcgaatgagagtttgatactttctttcgactttta gattggattttca
07970	NCgl1510	cacaccaattactcattctgcgatcccaaccgcttaaacggccgtgtaatggctttcccgcaatctagccgaattcgaccaaactaggtgaccgaaaacctccagccgaaaacctcaatggcaagcc caaactggtcacctggtttggtctgcactctgactcccctcaaaagggcacaatttggtcaatttcccaaccttgtctttcagtc
11080	NCgl2123	gcatcaatcttctttggcaacttcttttcgagcttgagttcagcgaccgttccacgaactggaagagtggcatccttggacaccttggttggtgctgttgtgctgtaggcatttttcgccattgaaagctga gtcctctcgttgaagttgtgtctccgctttggttgggggaggcatcaaattgaaactaacttttaacaagcctagccattcctcaaaaccgtgagacgaaattggctattcatcccataaaatggggctga ctagtgtatctgtcaggtagcaggtgtaccttaaaatcc
13230	NCgl2521	gcccacgatgtttaaataggcgatcggtgggcatctgtgtttggtttcgacgggctgaaaccaaaccagactgcccagcaacgacggaaatcccaaaagtgggcatccctgtttggtaccgagtacc cacccgggcctgaaactccctggcaggcgggcgaagcgtggcaacaactggaatttaagagcacaattgaagtcgcaccaagttaggcaacacaatagccataacgttgaggagttcag
10575	NCgl2022	aagttctcccttaaaaggttgaatcgaaattcttcgagctcggtgcccgaatccaagcggcctcaacacaagttcttaagcccgcatgtttacctattgtattccccacgtaacaagtttctgatttgggta catcagagttcatttgaattagacttaaaacttaaaatgaccaccccagatttacctgaattaaacccgctttcacctttgagatactggaagga
cysE	NCgl2474	gttaaccactcaagctctttgcttgggtggtttttcatgtctcaaggtcgggtcgggtgcgattcgggtcggttttgagtgtctttgagtccttttaagtccttctttgcccgtgaataattctctggatagtttc cacgtgcagttaagtcacgctgttagacttgcctgcatgctctcgacaataaaa

04295	NCgl0805	tttcttctcctaaaaagtgacacgggcgaatacaaatgggtgcgatatcgcaggtcaaacctcgacttttgctctgaaccaaggattgacttcacgctcttccccactgctgcaacgctggaattcaccg ctaacgatggctggattctcgagtctgtacacccttgatcaaagcccgagtgttccgtagattaactttgtcgtatattgtgacctacaccccatactgttaggagttttc
08980	NCgl1703	ttgttgaccactgacaactagcaagaagcggcaaatatgtgcggcttggcataaactgtgtccatcggtacctgtgtaatagacacacggtaaacacgcacaagataaaacattgcgagatttttcatg agtacaaaacccactatt
14180	NCgl2704	tgctgatgaactctgaaaccgtctcccgctggcgctaccttggcgttgccgaccgagaattcaaggcagagagctttgacaaccttcatgattttgaatctgacgcacttgacctactcattacagacg gtgagctccgcattgaacaagaacgcatccgcctcgatgttgctgttgaagaaatcgagaaagtaattcgtgggtgaagttgtttcagagttgtttttattaaacaactcagcctgtaacctcttggtcgtt ctatgcttggtcacc
eutD	NCgl2657	gggggtgaagagctgtaaagtaccgctaaaaactttgcaaagggtgcttcgcaacttgtaaccgctccgtattgttttctacggcaataagcatttgtgctgctcaaagcgtggaattgagatcggtttg aaaattacaaaataaaactttgcaaaccgggctgtacgcaaggcggacgaacgctaaactatgtaagaaatcacaacctcccctcattagtgccaggaggcacaagcctgaagtgtcatcaatgag aaggttcaggctgaaattagaaaggcgatgt
02840	NCgl0530	ccatctgcataaactaaaactgattccactgagtggaatctgaattcgtatcttcatctcttcttgacgacactttgagtcacacctaaaagtgatagccatcacgaatctttaggaaaagtgattcaaactt cactgtgatcggcttcggccacacacaagtgtcaggagatgaca
metX	NCgl0624	ctttaaatagactcaccccagtgcttaaagcgctgggtttttctttttcagactcgtgagaatgcaaactagactagacagagctgtccatatacactggacgaagttttagtcttgtccacccagaacag gcggttattttc

pyc	NCgl0659	cactcctgggttttcactttgttaagcagttttggggaaaagtgcaaagtttgcaaagtttagaaatattttaagaggtaagatgtctgcaggtggaagcgtttaaatgcgttaaacttggccaaatgtggc aacctttgcaaggtgaaaaactggggcggggttagatcctggggggtttatttcattcactttggcttgaagtcgtgcaggtcaggggagtgttgcccgaaaacattgagaggaaaacaaaaaccga tgtttgattgggggaatcgggggttacgatactaggacgcagtgactgctatcacccttggcggtctcttgttgaaaggaataattactcta
