# Supplementary material for: Obtaining a series of native gradient promoter-5′-UTR sequences in Corynebacterium glutamicum ATCC 13032
Source: Microb Cell Fact. 2020 Jun 3;19:120. doi: 10.1186/s12934-020-01376-3 (PMC7268698; doi:10.1186/s12934-020-01376-3)
Supplement: Supplementary file 2 — Additional file 2: Figure S1. Effects of typical chemicals on promoter strength. Figure S2. The level of inhibition or activation of PUTRs by typical chemicals.**: > 1 represent activation; < 1 repersents inhibition. [file 12934_2020_1376_MOESM2_ESM.pdf]

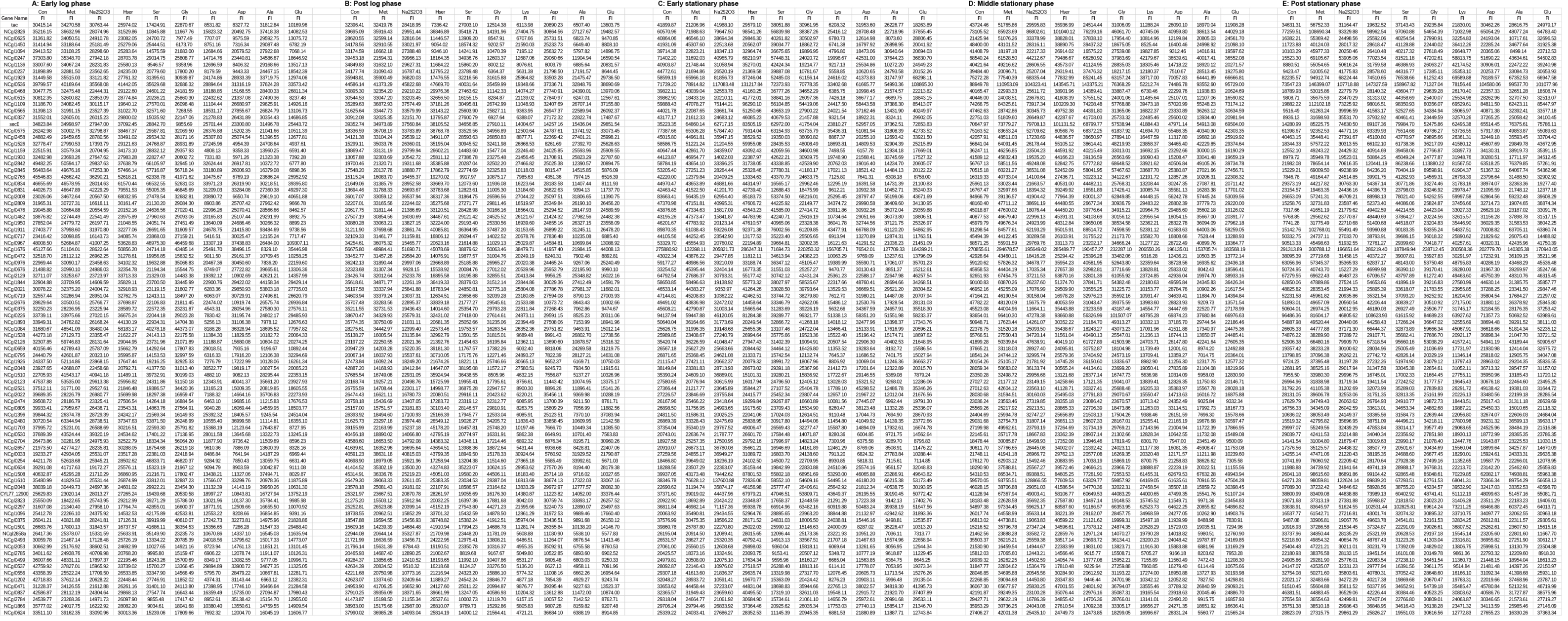

Figure S1. Effects of typical chemicals on promoter strength.

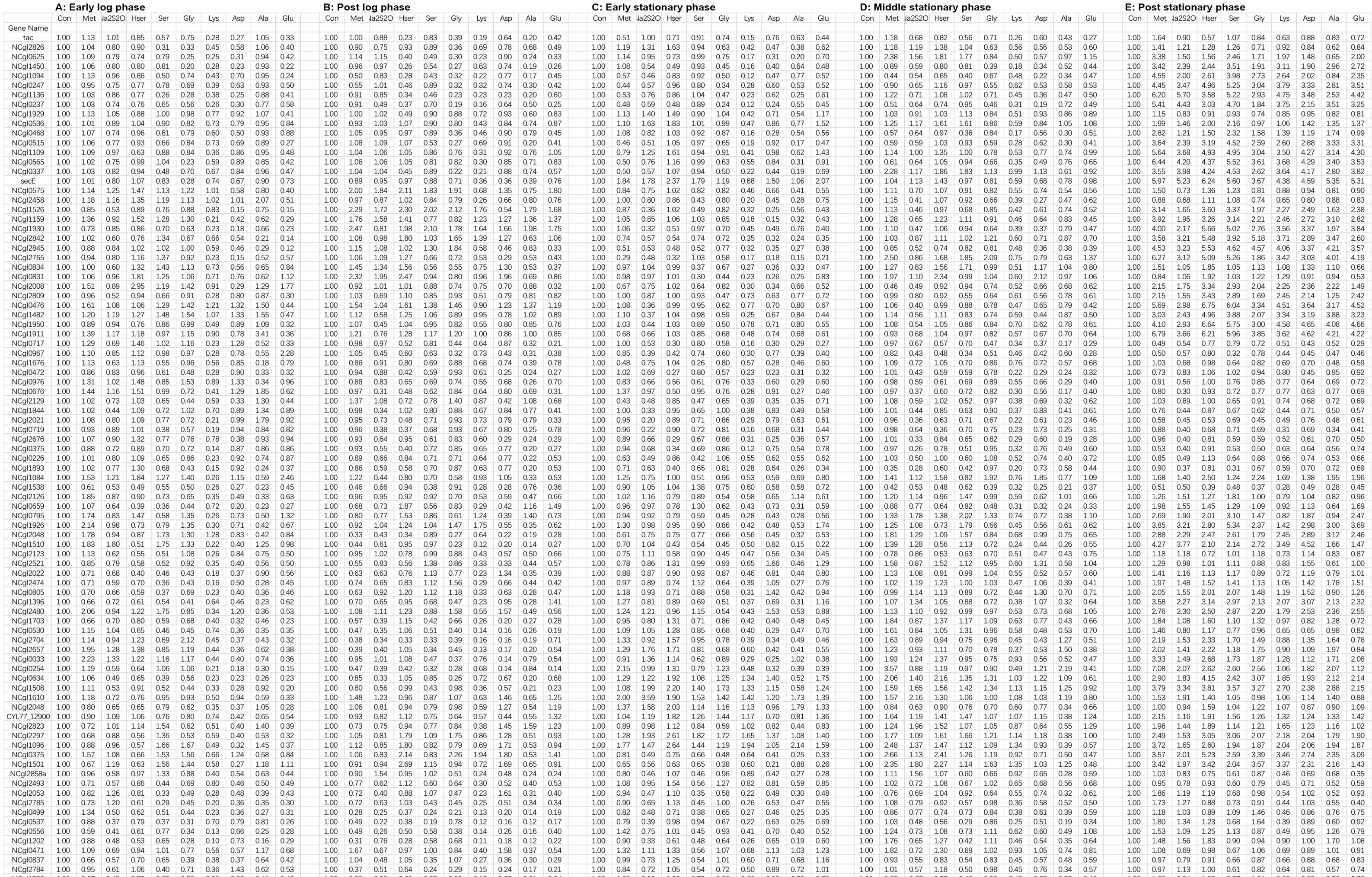

Figure S2. The level of inhibition or activation of PUTRs by typical chemicals. *

*: >1 represent activation; <1 represent inhibition
